# Tropical fish in a warming world: thermal tolerance of Nile perch *Lates niloticus* (L.) in Lake Nabugabo, Uganda

**DOI:** 10.1093/conphys/cow062

**Published:** 2016-12-15

**Authors:** Emmanuelle Chrétien, Lauren J. Chapman

**Affiliations:** Department of Biology, McGill University, Montreal, Quebec, Canada H3A 1B1

**Keywords:** Aerobic scope, body size, critical thermal maximum, freshwater fish, hypoxia tolerance, metabolic rate

## Abstract

Key to predicting the response of fishes to climate change is quantifying how close fish are to their critical thermal limits in nature and their ability to adjust their thermal sensitivity to maintain performance. Here, we evaluated the effects of body size and habitat on aerobic scope (AS) and thermal tolerance of Nile perch *Lates niloticus* (L.), a fish of great economic and food security importance in East Africa, using respirometry and critical thermal maximum (CTmax) trials. Juvenile Nile perch from distinct habitats (high or low dissolved oxygen concentrations) of Lake Nabugabo, Uganda were exposed for 4.6 ± 0.55 days to a temperature treatment (25.5, 27.5, 29.5 or 31.5°C) prior to experimentation, with the lowest temperature corresponding to the mean annual daytime temperature in Lake Nabugabo and the highest temperature being 3°C higher than the maximal monthly average. As expected, metabolic rates increased with body mass. Although resting metabolic rate increased with temperature, maximal metabolic rate showed no change. Likewise, AS did not vary across treatments. The CTmax increased with acclimation temperature. There was no effect of habitat on maximal metabolic rate, AS or CTmax; however, there was a trend towards a lower resting metabolic rate for Nile perch captured in the low-dissolved oxygen habitat than in well-oxygenated waters. This study shows that juvenile Nile perch maintain a large AS at temperatures near the upper limit of their natural thermal range and provides evidence that Nile perch have physiological mechanisms to deal with acute exposure to thermal stress.

## Abstract (precis)

Nile perch, a fish of food security importance in Africa, maintains a large aerobic scope at temperatures beyond the average temperature they encounter in the wild, suggesting they are not currently living at the edge of their thermal window and show capacity to adjust their thermal limits with acclimation.

## Introduction

Environmental temperature is a key predictor of species distribution that can affect the fitness and performance of individuals (Parmesan and Yohe, 2003; Pörtner and Farrell, 2008). Assuming organisms are adapted to the thermal regime of their environment, their temperature limits (upper and lower) and optimum should fall with the thermal range of their natural habitat (Janzen, 1967; Pörtner and Farrell, 2008). As such, tropical species are hypothesized to have narrow thermal windows relative to temperate species because they have evolved in relatively constant temperatures (Janzen, 1967). Such narrow thermal windows may pose a significant challenge in the face of predicted global temperature increases, because this may limit the range of thermal increase that permits persistence (Huey and Hertz, 1984). This is particularly true for ectotherms, such as fishes. In contrast to endotherms that maintain a constant internal body temperature through their metabolism, the body temperature of an ectotherm is a direct function of environmental temperature and is primarily controlled via behavioural thermoregulation (McNab, 2002). Therefore, the performance and fitness of ectotherms may be greatly influenced by variation in the environmental temperature. Tropical fishes (ectotherms that are likely to be thermal specialists) may then be particularly vulnerable to climate change (Tewksbury *et al*., 2008).

The ability of fishes to cope with rising water temperatures will depend on their capacity to shift to more favourable environments, acclimate via phenotypic plasticity and/or adapt through natural selection (Stillman, 2003; Perry *et al*., 2005; Ficke *et al*., 2007). In inland freshwater systems, fishes may have fewer options for dispersal to more thermally favourable environments because they are often trapped within landlocked water bodies and are more likely to have to respond *in situ* to climate warming. Thus, a mechanistic understanding of fish response to elevated water temperature and of the ability of freshwater fishes to adjust their thermal sensitivity are key to predicting the effects of climate change on fish populations (Stillman, 2003; Pörtner and Farrell, 2008).

The performance of ectotherms across a range of temperatures can be visualized as a bell-shaped—though not necessarily symmetrical—performance curve (referred to as a thermal window) with the efficiency of a given performance trait maximized at an optimal temperature (*T*_opt_) and falling to zero as species approach the upper (CTmax) and lower (CTmin) limits of their thermal window (Huey and Stevenson, 1979; Deutsch *et al*., 2008; Miller and Stillman, 2012). A key performance trait linked to metabolism and temperature is aerobic scope (AS). The metabolic scope for activity (or AS) was first proposed by Fry (1971) as a metric to assess an animal's ability to cope with environmental demands. Aerobic scope is an estimation of the metabolic energy available for activity (e.g. growth, reproduction) in a given environmental and physiological context, calculated as the difference between standard and maximal metabolic rates (SMR and MMR; Fry, 1971; Steinhausen *et al*., 2008; Pörtner, 2010). The SMR corresponds to the minimal metabolic rate required to sustain life, and the MMR is the maximal rate of oxygen consumption (Clark *et al*., 2013). Fry's paradigm suggests that environmental stressors shape an animal's activity through their effects on metabolism and classifies them into five categories based on their controlling, lethal, limiting, masking or directive effect on metabolism (Fry, 1971; Claireaux and Lefrançois, 2007). As such, water temperature is defined as a controlling factor because it sets the rate of metabolism, whereas dissolved oxygen (DO) availability will limit MMR (Claireaux and Chabot, 2016). Aerobic scope is hypothesized to be tightly linked to the thermal window and is likely to be maximal at the optimal temperature of the species, a theoretical framework known as oxygen- and capacity-limited thermal tolerance (OCLTT; Pörtner and Knust, 2007; Pörtner and Peck, 2010; Pörtner, 2010). Oxygen- and capacity-limited thermal tolerance predicts a decline in AS on both sides of the thermal window, as a result of decreased capacity of ventilatory and circulatory systems to cover physiological costs above maintenance levels, leading to hypoxaemia (Pörtner and Farrell, 2008; Pörtner, 2010). Beyond CTmin and CTmax, ectotherms transition to their passive, time-limited range of tolerance, an anaerobic mode of metabolism because of the critically low tissue oxygen levels (Pörtner, 2002).

Aquatic hypoxia is another potential stressor affecting the tolerance of fish to thermal stress. Dissolved oxygen availability strongly influences MMR (Fry, 1971), and thus hypoxia can reduce AS independent of temperature. Hypoxia and water temperature may also interact because both affect aerobic metabolism. By limiting the availability of environmental oxygen to fishes, hypoxia makes it more challenging to meet the increased metabolic demands driven by higher temperature (McBryan *et al*., 2016). However, adaptations to either thermal stress or hypoxia could improve the ability of fishes to cope with the alternative stressor (McBryan *et al*., 2016). Conceptual models predict that the tolerance to aquatic hypoxia in ectotherms is maximal within the thermal optimum and that processes that mitigate effects of thermally induced hypoxaemia should improve hypoxia tolerance (Pörtner, 2010). Conversely, tolerance to hypoxia may mitigate effects of thermal stress if cardiorespiratory systems in hypoxia-adapted species or populations are better able to deliver oxygen to the tissues than for fish from normoxic habitats. Thus, metabolic traits of fishes may vary among habitats characterized by divergent DO availability if fish are localized in distribution. Hence, characterizing habitat-specific variation in both oxygen availability and water temperature may be important in evaluating AS in fishes.

Fish body size is another factor likely to affect oxygen-limited thermal tolerance, given strong relationships between oxygen consumption and body size and metabolic costs associated with growth and reproduction. Larger fishes consume more oxygen per unit time, but less oxygen per unit mass per unit time. Therefore, total metabolic rate increases with body mass, whereas mass-specific metabolic rates decrease with mass (Clarke and Johnston, 1999; Gillooly *et al*., 2001; Chabot *et al*., 2016). Pörtner and Farrell (2008) proposed that thermal tolerance should be narrower for larval fish and spawners and widest in juveniles. The narrower range of temperature tolerance in larval fishes may be explained by their lower energy reserves despite their high mass-specific metabolic rates (Rijnsdorp *et al*., 2009), whereas in reproductive adults it is likely to be attributable to the increased oxygen demand to supply gonads (Pörtner and Farrell, 2008). Furthermore, thermal sensitivity is potentially higher in larger individuals (Rodnick *et al*., 2004; Pörtner and Knust, 2007), and the optimal temperature for growth and temperature preference have been shown to decline with body size (Björnsson *et al*., 2001; Lafrance *et al*., 2005; Rijnsdorp *et al*., 2009). Thus, in testing predictions related to OCLTT, intraspecific variation in body size is an important consideration.

The OCLTT concept has been successful in explaining the relationship between rising water temperatures and changes in the distribution and abundance of eelpout (*Zoarces viviparous*; Pörtner and Knust, 2007), in predicting spawning migration success in sockeye salmon (*Oncorhynchus nerka*; Farrell *et al*., 2008; Eliason *et al*., 2011) and in explaining counter-gradient variation in AS in some coral reef fishes (Nilsson *et al*., 2009). However, other studies have found that AS does not predict performance at elevated water temperatures (Norin *et al*., 2014). There is clearly a need for additional effort to evaluate the generality of the OCLTT hypothesis, particularly in tropical fishes, many of which contribute to regional food security. In this study, we evaluated the influence of body size and habitat on the AS and upper thermal tolerance of Nile perch [*Lates niloticus* (L.)] following a short-term acclimation to a range of elevated water temperatures on specimens collected from the field and tested near the site of capture.

### Study species and system

Inland fisheries represent one-third of total capture fisheries production in Africa, and for human populations living near the African Great Lakes (Victoria, Tanganyika and Malawi), fish protein is crucial, comprising an estimated 50% of per capita protein consumption (Food and Agriculture Organization of the United Nations, 2014). Lake Victoria, the largest tropical lake in the world, bordered by Uganda, Kenya and Tanzania, is home to Africa's largest inland fishery, composed primarily of the large piscivorous, non-native Nile perch (Balirwa *et al*., 2003). Nile perch were introduced to Lake Victoria and other lakes in the region (Nabugabo and Kyoga) in the 1950s and 1960s to compensate for depleting commercial fisheries (Balirwa *et al*., 2003; Pringle, 2005). The exponential growth of the Nile perch population in Lake Victoria during the 1980s resulted in the rapid development of a new fishing industry and a very important export market but also contributed (at least in part) to the decline or loss of many native fishes, including the disappearance of ~40% of endemic haplochromine cichlids (Witte *et al*., 1992a, b; Kaufman and Ochumba, 1993; Seehausen and Bouton, 1997). Collapse of the native fish community and emergence of Nile perch as a dominant fish stock also occurred in other lakes in the region, including lakes Kyoga and Nabugabo (Ogutu-Ohwayo, 1993, 1990). However, sustained high fishing pressure on Nile perch has resulted in a decline in yields in Lake Victoria and Lake Nabugabo, and some native species that were in decline have resurged (Balirwa *et al*., 2003; Ogutu-Ohwayo, 2004; Witte *et al*., 2007; Paterson and Chapman, 2009; Taabu-Munyaho, 2014). Although this is positive for the persistence of native species, there are serious concerns about the sustainability of the Nile perch fishery, and there is great interest in predicting the response of Nile perch to environmental stressors, including elevated water temperature associated with climate warming (Paterson and Chapman, 2009; Nyboer and Chapman, 2013a). Selection by Nile perch for well-oxygenated, cool waters (Nyboer and Chapman, 2013a) and its high resting rate of oxygen consumption (Schofield and Chapman, 2000; Chapman *et al*., 2002) suggest that Nile perch may be particularly sensitive to thermal stress.

Our study was conducted on Nile perch collected from Lake Nabugabo, Uganda between June and August 2014. Formerly a bay of Lake Victoria, Lake Nabugabo became isolated from the main lake ~5000 years ago (Stager *et al*., 2005). It is a much smaller and more shallow lake (surface area 33 km^2^, mean depth 3.13 m; Nyboer and Chapman, 2013a) than Lake Victoria. Dense wetlands structure most of the littoral zone of the lake, except for the west side that is bordered by forest and fishing villages. Wetland ecotones are characterized by lower DO concentrations than the forest edge and by dense structure from both submerged and emergent macrophytes (Schofield and Chapman, 1999; Paterson and Chapman, 2009). There is evidence for ecological divergence in juvenile Nile perch associated with these distinct habitats, which may reflect contrasting DO concentration, prey availability and/or other differences between the two habitats. For example, juvenile Nile perch captured in or near wetland ecotones are characterized by larger gill size (Paterson *et al*., 2010), an earlier shift to a piscivorous diet (Paterson and Chapman, 2009) and a different body shape (Nyboer and Chapman, 2013b) and colour (Nyboer *et al*., 2014) than juvenile perch from well-oxygenated inshore waters. As a result of the distinct environmental conditions between habitats and the high site fidelity of the species (Nyboer and Chapman, 2013a), thermal tolerance of Nile perch may also be expected to differ. As such, we predicted a narrowing in AS with increasing body size and temperature, and a wider AS for individuals showing evidence for adaptation to hypoxia (Pörtner *et al*., 2008; Pörtner, 2010; McBryan *et al*., 2013).

## Materials and methods

**Figure 1:**
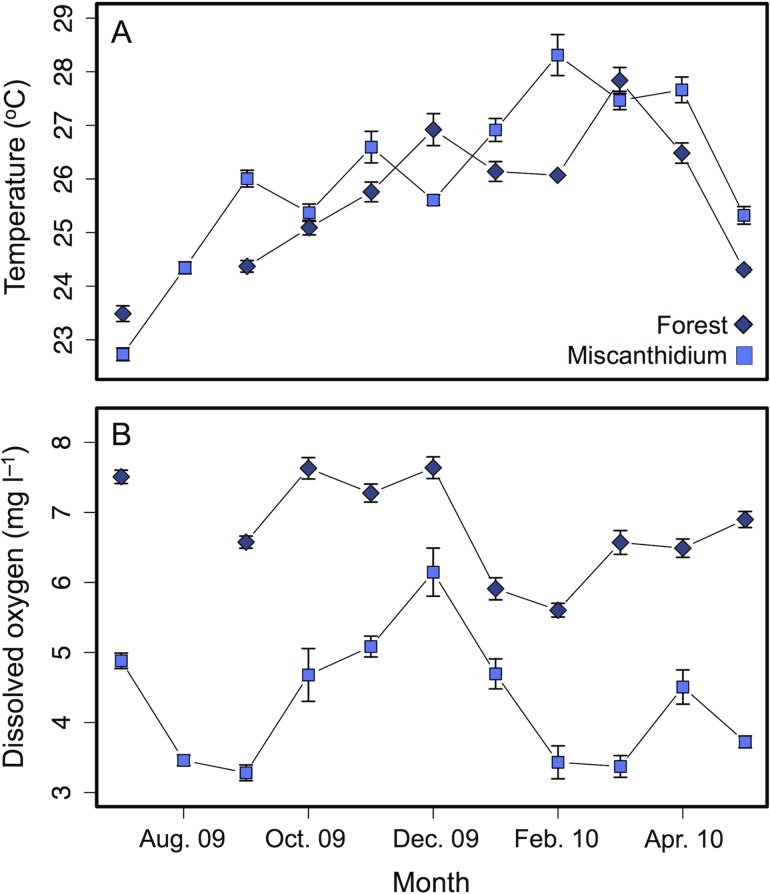
Mean daytime temperature (**A**) and dissolved oxygen concentrations (**B**) of the water column in Forest (diamonds) and Miscanthidium (squares) habitats of Lake Nabugabo. Measurements were taken monthly from July 2009 to May 2010 across four sites in the littoral zone (within 20 m from the shore) of each habitat (except for August 2009 in Forest).

Environmental data collected from July 2009 to May 2010 revealed two Lake Nabugabo habitats similar in thermal regime but divergent in DO concentrations: the wetland ecotone bordered by *Miscanthidium violaceum*, ‘Miscanthidium’; and the forest edge, ‘Forest’ (Chrétien and Chapman, 2016). Throughout the 11 month study, the two habitats showed similar mean annual daytime temperature but contrasting DO concentration (Fig. 1 and Table 1). The present study was conducted on Nile perch captured live in Miscanthidium and Forest habitats, because DO concentration may be a factor contributing to differences in oxygen uptake capacity and therefore the ability of Nile perch to perform near their upper thermal window.

**Table 1:** Mean daytime temperature (in degrees Celsius) and dissolved oxygen concentrations (in milligrams per litre) in habitats of Lake Nabugabo (Uganda) under study

Measurement	Temperature (°C)		Dissolved oxygen (mg l^−1^)	
	Forest	Miscanthidium	Forest	Miscanthidium
July 2009 to May 2010	25.65 ± 0.18	26.03 ± 0.21	6.81 ± 0.11	4.30 ± 0.17
Summer 2009	23.49 ± 0.39	23.54 ± 0.45	7.51 ± 0.25	4.17 ± 0.38
Summer 2014	24.01 ± 0.34	23.73 ± 0.14	6.89 ± 0.14	3.73 ± 0.57

Means from the year 2009–10 and summer 2009 were measured monthly across four sites in the littoral zone of Miscanthidium and Forest edge habitats in Lake Nabugabo. Means from summer 2014 were measured at each sampling effort. Values are presented as means ± SEM.

### Fish sampling

Nile perch were captured at dusk or dawn by beach seines in Miscanthidium or Forest habitat. A maximum of eight fish were brought back to the field station facilities at one time, to minimize holding densities, and placed in one of the five holding tanks (three 75 litre and two 109 litre tanks) supplied with Nabugabo lake water for 24 h to recover from capture before acclimation to a target temperature. After the 24 h recovery period, the water temperature was increased with aquarium heaters to one of four acclimation temperature treatments (which occurred over a maximum of 12 h; 25.5, 27.5, 29.5 or 31.5°C) determined based on the lake thermal regime and estimated increases in temperatures associated with climate change. Air temperature for the Lake Victoria basin is predicted to increase by 4°C before 2100, based on a Representative Concentration Pathway (RCP8.5) model (Niang *et al*., 2014). The lowest acclimation temperature treatment of 25.5°C was determined based on Lake Nabugabo mean annual daytime water temperatures in 2009–10 (Table 1), and the highest acclimation temperature, 31.5°C, is 2°C above the increase predicted by the RCP8.5 model.

Dissolved oxygen was maintained at air saturation with air bubblers, and papyrus (*Cyperus papyrus*) flowers were put in tanks to provide cover. A 50% water replacement was done every morning, and tanks were refilled with lake water treated with NovAqua (5 ml l^−1^). Fish were fed Ephemeroptera (mayfly) larvae collected in the lake or Tetramin tropical fish flakes (depending on availability of live food) daily during the acclimation period, but were starved for 24 h prior to experiments to ensure a post-absorptive state and prevent bias in measurements. Unconsumed food was removed from the tanks at water changes.

Two trial types were conducted: respirometry and CTmax. Respirometry trials included fish from all temperature treatments, and CTmax trials were performed on fish held at 25.5, 27.5 and 29.5°C for a minimum of 3 days. Exposure time to the temperature treatments averaged 4.9 ± 0.57 days (range 3–12 days) for respirometry trials and 4.3 ± 0.52 days (range 3–13 days) for CTmax trials. The logistics of capturing and holding fish at the field site and availability of power for acclimation contributed to the variability around the average exposure times, which we address in our results. No fish died during experiments. This research was conducted with the approval of McGill University's Animal Care Committee (AUP 5029) and Uganda National Council for Science and Technology.

### Metabolic rates

The minimal metabolic rate (SMR) is typically measured on post-absorptive fish and estimated as the lowest rate reached over a full diel cycle (Clark *et al*., 2013). We chose to measure resting metabolic rate (RMR) as a best estimate of the SMR of Nile perch because field logistics (availability of continuous power) did not allow for acclimation times longer than a few hours in the respirometry chamber, ruling out the possibility of recording full diel cycles in the respirometer. Thus, the calculation of the difference between MMR and RMR provides us with an underestimation of AS, used here as a proxy for the true AS.

Each experiment consisted of an RMR trial followed by an MMR trial. Measurements of metabolic rates were made using intermittent respirometry controlled manually with a Loligo Witrox 1 oxygen meter for mini sensors and WitroxView software (Loligo Systems, Tjele, Denmark). Two independent systems were placed in a large cooler (filled with 46 litres of lake water), allowing two fishes to be tested simultaneously. Each system consisted of a chamber connected to two circuits of water tubing and flush pumps: an open circuit, allowing water exchange with the basin; and a closed circuit, recirculating water in and out of the chamber (see Supplementary Fig. 1). When the open circuit was turned off, the DO concentration in the chamber declined via respiration of the fish. Visual stimulus that could be induced by other Nile perch was limited because of the position of the chambers and presence of equipment between them. Four chambers were used, with volume capacity ranging from 0.48 to 3.42 litres depending on fish size; producing an average fish mass-to-respirometer volume ratio of 1:77. The DO in the basin was maintained at above 95% air saturation by air bubblers, and water temperature was kept constant by aquarium heaters.

Fish were placed in their respirometry chamber and acclimated to the system for 2 h before initiating the trial, consistent with previous respirometry experiments conducted at the Nabugabo field station (Schofield and Chapman, 2000; Reardon *et al*., 2011; McDonnell and Chapman, 2015). During this time, the length of the measurement period was determined by observing how rapidly oxygen was declining in the chamber. Measurement periods would generally be 10 min but were cut to 5 min if DO concentrations were likely to go below 80% air saturation. Flush periods lasted 15 min. During acclimation and throughout the trial, the switch between measurement and flush periods was done manually by turning power off or on. After the acclimation period, four measurements of oxygen consumption (the slope of depletion of oxygen concentration with time) were recorded. The RMR of fish was obtained by calculating the average from these four measurements of rate of oxygen consumption. In general, the metabolic rate of the Nile perch declined during the acclimation period, stabilizing after ~90 min. It is possible that the metabolic rate of Nile perch may reach lower levels during a 24 h cycle; however, our consistent pre-test period permitted robust comparisons of response variables among fish from different habitats and across a range of body sizes.

After recording oxygen consumption for calculation of RMR, fish were taken out of the chamber and subjected to a standardized protocol to induce exhaustion of the fish. The MMR was estimated as the rate of oxygen consumption of the Nile perch after an exhaustive 3 min chase protocol followed by 1 min air exposure, a reliable alternative method to swimming respirometry (Roche *et al*., 2013). The MMR trial consisted of four measurement periods. The MMR was the single highest rate of oxygen consumption value recorded, and generally corresponded to the first measurement after the chase protocol. Before and after each respirometry trial, the background respiration of each empty chamber was quantified by a single long measurement (minimum of 20 min).

### Critical thermal maximum

The upper critical thermal tolerance (CTmax) of Nile perch was quantified following a methodology adapted from Fangue *et al*. (2006) and Chen *et al*. (2013). Fish were challenged individually in a 25 litre glass aquarium inserted in a larger glass aquarium (100 litres), both of which were filled with lake water maintained at the fish treatment temperature. The outer aquarium served to insulate the trial tank, ensuring maintenance and steady increase of water temperature through the experiment. The trial tank was kept near 100% air saturation with air bubblers. After transfer to the trial tank, fish were given 2 h at their acclimation temperature to recover from the stress of transfer. The water temperature was then increased in the trial tank at a rate of 1°C every 3 min by a slow flow of hot water from a reservoir over the tank. The water level in the trial tank was maintained constant by an outflow. The CTmax was defined as the temperature at which fish lost equilibrium, at which point fish were removed from the trial tank and transferred to a holding tank at their acclimation temperature to recover. Throughout trials, the DO concentration and water temperature were recorded at 90 s intervals, and the rate of temperature increase was calculated for each trial (average rate of increase: 0.329 ± 0.004°C min^−1^). An increase of 0.3°C min^−1^ is unlikely to occur in nature; however, this rate is commonly used in CTmax studies because it is fast enough to prevent fish gaining thermal tolerance during the trial and slow enough for the fish's body temperature to change with the water temperature (Becker and Genoway, 1979; Beitinger *et al*., 2000).

### Data analysis

A total of 68 Nile perch were used for the respirometry and CTmax trials (Supplementary Table 1). The final data set for RMR analysis included 34 Nile perch averaging 13.0 ± 0.7 cm in total length (TL; range 6.2–19.4 cm) and 24.8 ± 2.8 g in body mass (range 2.4–53.7 g). The CTmax was estimated for 34 Nile perch averaging 12.1 ± 0.8 cm in total length (range 6.5–22.2 cm) and 26.7 ± 4.9 g in body mass (range 2.6–113.2 g).

Metabolic rates were calculated from the slope of decline in oxygen concentration during measurement segments, excluding the first minute after closing the valve and the minute before reopening it. The oxygen concentration measurements (DO; in milligrams per litre) were converted to metabolic rate (MO_2_; in milligrams per minute) using the following equation:

(1) $${\mathrm{MO}}_{2}=\left|∆\mathrm{DO}/∆t\right|\times \mathrm{vol}\;\times \;60,$$

where *∆*DO*/∆t* is the slope of oxygen decrease over time (in seconds), and vol is expressed in litres and corresponds to the volume of water in the closed system (chamber, pump and water tubing) corrected for the volume of the fish (assuming a density of 1 kg l^−1^). Background oxygen consumption was subtracted from MO_2_. If background oxygen consumption increased during the trial, fitted background respiration values obtained from the linear regression between empty chamber oxygen consumption values (before and after) and time were subtracted from MO_2_. The MO_2_ values for which background respiration represented >25% of RMR were removed from the data set. Values of MMR were excluded when they did not exceed the maximal rate obtained during acclimation of the fish.

Aerobic scope was calculated in two ways: absolute aerobic scope (MMR − RMR) and factorial aerobic scope (MMR/RMR). The former is an indication of the absolute increase in oxygen consumption rates above minimal levels, and the latter indicates the factorial increase (Clark *et al*., 2013). Mass-adjusted metabolic rates (in milligrams per minute per kilogram) were used for calculation of *Q*_10_ values and calculated using the following equation (Steffensen *et al*., 1994; Ultsch, 1995):

(2) $$\begin{array}{c} {\mathrm{MO}}_{2\mathrm{adj}}={\left(\mathrm{mean mass of fishes}\right)}^{b-1}\times {\left(\mathrm{observed mass}\right)}^{1-b}\\ \times {\mathrm{observed MO}}_{2}, \end{array}$$

where *b* is the slope of the log–log relationship between metabolic rate and body mass (*b*_RMR_ = 0.8507; *b*_MMR_ = 0.8357; Supplementary Table 2), mass values are expressed in kilograms, and observed MO_2_ values are MO_2_ values obtained by equation 1 divided by the mass of the fish, and are therefore expressed in milligrams per minute per kilogram.The *Q*_10_ coefficients, representing the sensitivity of an organism to temperature fluctuations, are a measurement of the change in metabolic rate over a 10°C range. The *Q*_10_ values were calculated from mass-adjusted RMR and MMR mean values, where MO_2,1_ and MO_2,2_ are metabolic rates at temperatures *T*_1_ and *T*_2_, respectively (McNab, 2002).

(3) $$Q_{10}=({\mathrm{MO}}_{2,2}/{\mathrm{MO}}_{2,1})\;\times \;[10/\left(T_{2}-T_{1}\right)]$$

### Statistical analysis and calculations

All analyses were performed in R v3.1.2 (R Development Core Team, 2014). Analysis of covariance (ANCOVA) was used to test the effects of habitat, experimental temperature and their interaction on RMR, MMR and AS, using fish body mass as a covariate. Mean values were adjusted to a common body mass and common regression line from the ANCOVA models. Acclimation time and its interaction with the other effects were also included in initial models as a second covariate because it ranged from 3 to 12 days. Non-significant interaction terms were sequentially removed from the models. The covariate acclimation time was added to the models as a precautionary measure, in case it contributed to variation in the response variables, but it was then removed from models because: (i) its effect and interaction with other effects were non-significant; (ii) its removal improved (or did not significantly change) the fit of the model using Akaike information criterion (AIC); and (iii) regression of residuals from the final models against acclimation time did not yield significant results. An effect of sex was not included because Nile perch reach sexual maturity at a much larger size than the range covered with this study (>50 cm TL in males and >80 cm TL in females; Ogutu-Ohwayo, 2004). As the relationship between metabolic rate and fish mass is allometric (McNab, 2002), those variables were log_10_ transformed to linearize relationships. The CTmax data were first analysed using ANCOVA with fish TL, body mass or acclimation time as covariates. Neither covariate was significant, thus a factorial analysis of variance (ANOVA) was used to assess the effects of habitat, experimental temperature and their interaction on CTmax. Pairwise comparisons were calculated using Tukey's test (*lsmeans* package in R; Lenth and Hervé, 2015). Unless stated, values presented are means ± SEM.

## Results

### Metabolic rates

**Figure 2:**
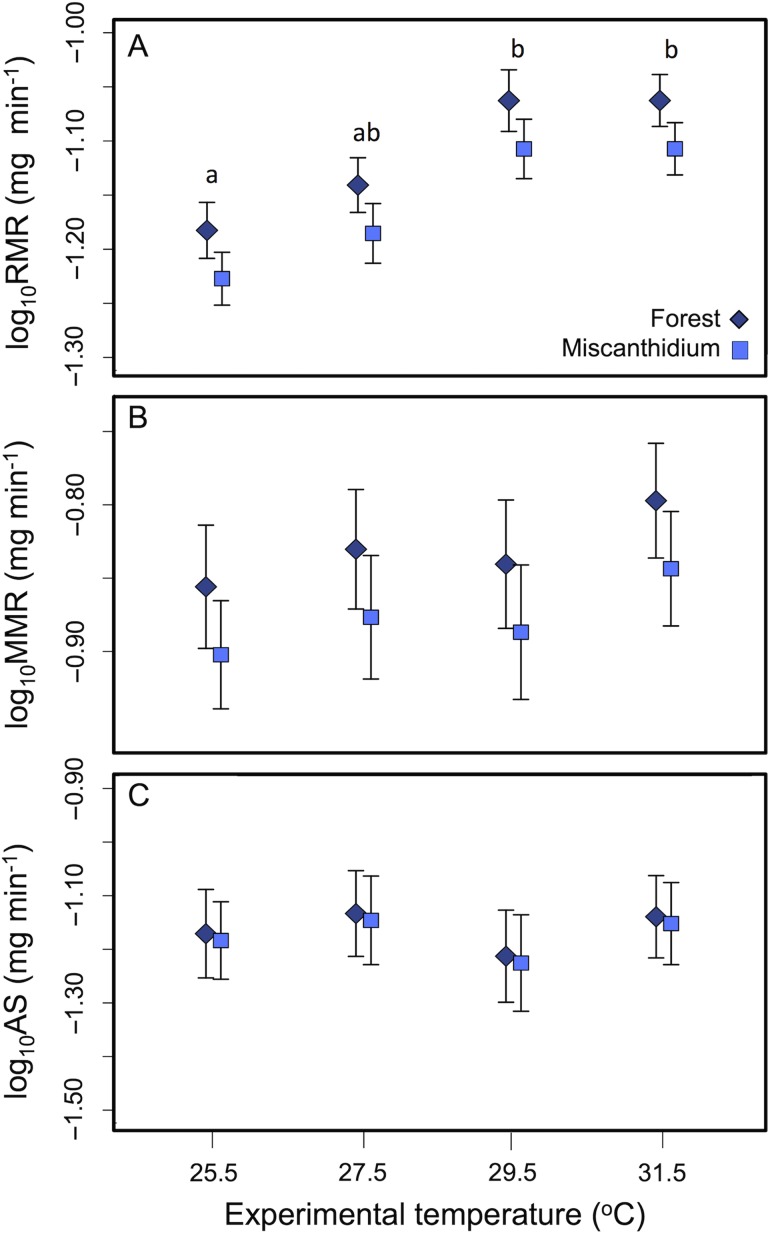
Log_10_ mean resting metabolic rate (RMR; **A**), maximal metabolic rate (MMR; **B**) and aerobic scope (AS; **C**) of Nile perch captured in high-dissolved oxygen (Forest; diamonds) and low-dissolved oxygen habitat (Miscanthidium; squares) acclimated to four experimental temperatures. Means are adjusted to the common log_10_ mean body mass (RMR, 17.2 g, *n* = 34; and MMR and AS, 17.0 g, *n* = 31) with standard errors (±1SEM) calculated from analyses of covariance. Letters indicate significant differences where present in mean RMR across experimental temperatures determined from *post hoc* tests.

Average metabolic rate measurements (expressed in milligrams per minute per kilogram) for Nile perch collected from the two habitats and acclimated to four temperatures are reported in Table 2. Experimental temperature had a significant effect on RMR (*P* = 0.001; Table 3). The average RMR of Nile perch acclimated to 29.5 and 31.5°C was higher than the RMR of Nile perch acclimated at 25.5°C (Fig. 2A). In addition, there was a trend towards a lower average RMR for Nile perch captured in Miscanthidium than for those captured in Forest (*P* = 0.065; Table 3 and Fig. 2A).

**Table 2:** Average metabolic rates (expressed in milligrams per minute per kilogram) of Nile perch by habitat of capture and experimental temperature (in degrees Celsius)

Habitat	Temperature (°C)	RMR (mg min^−1^ kg^−1^)	MMR (mg min^−1^ kg^−1^)	AS (mg min^−1^ kg^−1^)
Forest	25.5	3.81	8.39	4.15
	27.5	4.19	9.03	4.64
	29.5	5.02	8.79	3.77
	31.5	5.02	9.73	4.46
Miscanthidium	25.5	3.44	7.59	3.99
	27.5	3.78	8.17	4.45
	29.5	4.53	7.95	3.62
	31.5	4.53	8.80	4.28

Abbreviations: AS, aerobic scope; MMR, maximal metabolic rate; and RMR, resting metabolic rate.

**Table 3:** Results of analyses of covariance conducted to detect effects of acclimation temperature, habitat and their interaction on resting metabolic rate (RMR), maximal metabolic rate (MMR) and aerobic scope (AS), with Nile perch body mass as a covariate

Model	Effect	*F*	d.f.	*P*-value
Log_10_RMR	Log_10_Mass*	906.43	1, 28	<0.001
	Experimental temperature*	6.81	3, 28	0.001
	Habitat	3.69	1, 28	0.065
Log_10_MMR	Log_10_Mass*	373.99	1, 25	<0.001
	Experimental temperature	0.77	3, 25	0.519
	Habitat	1.50	1, 25	0.232
Log_10_AS	Log_10_Mass*	96.83	1, 25	<0.001
	Experimental temperature	0.08	3, 25	0.783
	Habitat	0.30	1, 25	0.822

Non-significant interaction terms were removed from models. Response variables and body mass were log_10_ transformed. *Significant terms.

The MMR did not vary with temperature or habitat (Table 3 and Fig. 2B). Likewise, AS did not vary across temperature treatments or habitats (Table 3 and Fig.2C). No model using the factorial aerobic scope (FAS) as the response variable was significant.

The *Q*_10_ value was 1.54 for RMR and 1.33 for MMR over the entire 6°C experimental temperature range (25.5–31.5°C). The *Q*_10_ values for RMR ranged from 1.68 between 25.5 and 27.5°C to 2.14 between 27.5 and 29.5°C, and dropped to 1.02 between 29.5 and 31.5°C. The *Q*_10_ values for ranges where significant differences in RMR were detected were 1.89 (25.5–29.5°C) and 1.54 (25.5–31.5°C). The *Q*_10_ values for MMR were 1.56 between 25.5 and 27.5°C, 0.86 between 27.5 and 29.5°C and 1.78 between 29.5 and 31.5°C, although it should be noted that the differences in MMR were not significant across temperature treatments.

### Critical thermal maximum

**Figure 3:**
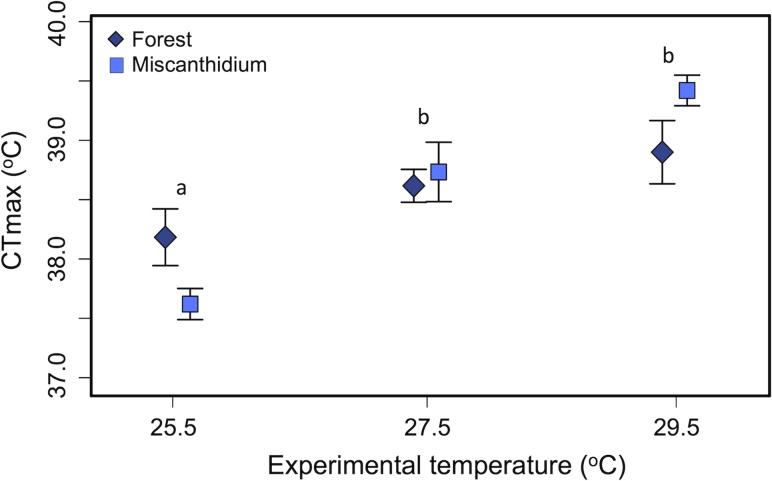
Mean (±1SEM) critical thermal maximum (CTmax) temperatures of Nile perch captured in high-dissolved oxygen (Forest; diamonds) and low-dissolved oxygen habitat (Miscanthidium; squares) acclimated to three experimental temperatures. Letters indicate significant differences across experimental temperatures determined from *post hoc* tests.

Average CTmax was 38.58 ± 0.26°C. The CTmax increased with acclimation temperature but did not differ between Nile perch from different habitats (*P* < 0.001 and *P* < 0.901, respectively; Table 4 and Fig. 3). There was no interaction between temperature treatments and habitat (*P* = 0.13). The CTmax for individuals acclimated to 25.5°C was lower than for Nile perch acclimated to 27.5 or 29.5°C (Table 4 and Fig. 3). Average CTmax for juvenile Nile perch increased by 1.2°C between 25.5 and 29.5°C groups, with a slope relating CTmax to acclimation temperature of 0.30 ± 0.07.

**Table 4:** Effects of acclimation temperature, habitat and their interaction with critical thermal maximum (CTmax) of Nile perch

Model	Effect	*F*	d.f.	*P*-value
CTmax	Experimental temperature*	10.15	2, 30	<0.001
	Habitat	0.02	1, 30	0.901

Non-significant interaction terms were removed from models. *Significant terms.

### Acclimation time

Although acclimation time was not a significant factor in our models, given the range in acclimation times, we repeated the statistical analyses on subsets of respirometry and CTmax trial data sets excluding fish acclimated for 7 days or more. Results for RMR, MMR and AS did not change. Results from CTmax analysis showed a minor difference, in that average CTmax for individuals acclimated to 25.5°C was not different from that of those acclimated to 27.5°C. Results from the reduced data set are reported in Supplementary Tables 3 and 4.

## Discussion

For this study, juvenile Nile perch were exposed to a range of biologically relevant temperatures, with the lowest acclimation treatment corresponding to the mean annual daytime temperature in Lake Nabugabo (25.5°C) and the highest temperature (31.5°C) being 3°C more than the maximal monthly average. The highest acclimation temperature was 0.1°C higher than the maximal temperature measured in Lake Nabugabo in 2009–10 (afternoon measurement in Miscanthidium inshore site, February 2010, 31.4°C; Chrétien, 2015), and represents a temperature that may occasionally be experienced by Nile perch. Juvenile Nile perch maintained a large AS at temperatures at the upper limit of their natural range, and CTmax results provide evidence that Nile perch can adjust their thermal sensitivity through acclimation, although not sufficiently to match the increase in acclimation temperature.

### Nile perch aerobic performance

The RMR increased with temperature, as expected, because rates of biochemical reactions are temperature dependent (Gillooly *et al*., 2001). As water temperature increases, so should the minimal energy needed to maintain homeostasis, which therefore increases metabolic rates (Brett, 1971). As such, increases in RMR (or SMR) with temperature have been reported for a diversity of both temperate (Brett, 1971; Steinhausen *et al*., 2008; Fangue *et al*., 2009; Healy and Schulte, 2012) and tropical freshwater and marine fish species (Fernandes and Rantin, 1989; Barrionuevo and Fernandes, 1998; Nilsson *et al*., 2009; Donelson and Munday, 2012; Norin *et al*., 2014). Routine metabolic rate did not, however, increase significantly between 29.5 and 31.5°C, which is reflected by the *Q*_10_ value of 1.02. The same pattern was observed in the common killifish (*Fundulus heteroclitus*) thermally acclimated for 4 weeks; routine metabolic rate increased with acclimation temperature from 5 to 25°C and then plateaued in the highest temperature treatments of 30 and 33°C (Healy and Schulte, 2012). This result may reflect thermal acclimation abilities. Fish held in the highest temperature treatment could also have entered a hypometabolic state (lowered their metabolic rates) to cope with sustained exposure to stressful environmental conditions (Storey and Storey, 2004).

The MMR of juvenile Nile perch did not vary across temperature treatments. Likewise, the MMR of three damselfishes and one cardinalfish (*Dascyllus anuarus*, *Chromis atripectoralis*, *Acanthochromis polyacanthus* and *Ostorhinchus doederleini*) acclimated for 1 week to a range of temperatures between 29 and 33°C did not vary (Nilsson *et al*., 2009). Donelson and Munday (2012) also observed no difference in MMR in *A. polyancanthus* when acclimated (27–31.5°C; 28 month acclimation) or acutely exposed (from 27 to 30°C, or from 28.5 to 31.5°C; 1 week acclimation) to warmer temperatures. In a widespread African cichlid, *Pseudocrenilabrus multicolor victoriae*, acclimated for 1 week to temperatures between 24 and 34°C, MMR increased with temperature and then plateaued (McDonnell and Chapman, 2016). Other studies have observed a decline in MMR in high temperature treatments. When acutely exposed to increasing water temperatures (from 15 to 24°C; increase of 2°C h^−1^), the MMR of sockeye salmon (*Oncorhynchus nerka*) increased to its highest level at 23°C, and decreased at 24°C (Steinhausen *et al*., 2008). Likewise, MMR increased with temperature until 30°C and then declined at 33°C in a northern population of *F. heteroclitus* acclimated to different temperature treatments (5–33°C; 4 week acclimation; Healy and Schulte, 2012), whereas for *F. heteroclitus* acutely exposed to higher temperatures (from 15 to 20, 25, 30 or 33°C; 15 min), MMR increased and then plateaued. Contrasting results were observed in a congener of Nile perch, the barramundi *Lates calcifer*. The MMR increased continuously in *L. calcifer* acutely exposed to warmer temperatures (from 29 to 35 or 38°C; increase of 2°C h^−1^), whereas it did not vary in thermally acclimated barramundi (29 or 38°C; 5 week acclimation; Norin *et al*., 2014).

### Testing the OCLTT hypothesis

As a result of the hypothesized overlap between an organism's thermal window and the thermal range of its natural habitat (Janzen, 1967; Pörtner and Farrell, 2008), *T*_opt_ and average habitat temperature might be expected to be similar. The OCLTT hypothesis predicts a decline in AS at temperatures higher than *T*_opt_ because MMR reaches its limit, whereas RMR continues to rise (Pörtner, 2010). We did not observe a decrease in AS with temperature in the present study, but we did observe an underlying pattern consistent with the OCLTT framework; there was an increase in RMR with temperature, whereas MMR remained unchanged. Heart rate is expected to be maximal at *T*_opt_ in active fish (i.e. at MMR; Farrell, 2009). Therefore, MMR is not expected to continue to increase at temperatures above *T*_opt_ as the heart reaches its maximal capacity to deliver oxygen to the tissues (Pörtner, 2010), which should lead to a decline in AS. However, the absence of significant variation in AS that we observed across the range of temperature treatments may also reflect the breadth of *T*_opt_. The OCLTT concept predicts maximal AS to be reached at *T*_opt_, but performance can remain optimal over a range of temperatures around *T*_opt_ (*T*_opt_ window), also defined as thermal performance breadth (Huey and Stevenson, 1979). Thus, the stability in AS that we observed may be consistent with the OCLTT hypothesis, if the temperatures tested were encompassed within the *T*_opt_ window for juvenile Nile perch in Lake Nabugabo. Mean daily temperatures in the lake ranged from 22.2 to 28.6°C in 2009–10 (Chrétien and Chapman, 2016), which could indicate that the range of temperature treatments (25.5–31.5°C) did not exceed the juvenile Nile perch *T*_opt_ window.

The OCLTT hypothesis has been widely applied to predict the response of fish to elevated temperatures; however, empirical support for the framework is mixed, which may reflect both interspecific variation in thermal ecology and/or contrasting protocols (fish thermally acclimated or acutely exposed to experimental temperatures). In a study of AS of five coral reef fishes acclimated to different temperatures (29–33°C; 1 week acclimation), Nilsson *et al*. (2009) found a decline in AS with temperature in all but one species, a pattern generally consistent with the OCLTT hypothesis. In contrast, Clark and colleagues (2011) measured the highest AS at a temperature higher than *T*_opt_ in pink salmon (*O. gorbuscha*) acutely exposed to increasing temperatures (from 13 to 24°C; increase of 2°C h^−1^). Furthermore, the AS of the barramundi (*L. calcifer*) acutely exposed to higher temperatures (from 29 to 35 or 38°C; increase of 2°C h^−1^) increased constantly up to 38°C, a temperature close to their upper critical thermal limit, whereas the AS of individuals thermally acclimated to 29 and 38°C [5 week acclimation] were not significantly different (Norin *et al*., 2014). Warm-acclimated fish are likely to reduce their metabolic machinery to conserve energy, limiting MMR and thus reducing AS, as the capacity for high MMR is costly and not required for regular activities (Norin *et al*., 2014). In our study, the exposure time was short, and this may have affected the ability of Nile perch to acclimate thermally to the temperature treatments. However, it is interesting that the pattern of response in RMR, MMR and AS that we observed for Nile perch was similar to results for estimates of SMR, MMR and AS in the congener, *L. calcifer*, acclimated for 5 weeks to elevated temperature (Norin *et al*., 2014). Barrionuevo and Fernandes (1998) reported that full acclimation to a higher temperature takes a week or less for most species, and occurred after 1–3 days in the South American freshwater fish *Prochilodus scrofa*. Furthermore, *Q*_10_ values for animals that show acclimation to different temperatures are generally <2, and between 2 and 3 when acutely exposed (Tullis and Baillie, 2005). Changes in RMR and MMR of juvenile Nile perch across temperatures were 1.53 and 1.33, respectively, values indicating that fish metabolically compensated to experimental temperatures. Certainly, there might be other responses of Nile perch to thermal stress if a longer acclimation time were to be used, if they were to be reared at elevated temperatures (via developmental plasticity), or if selection for thermal compensation resulted in genetic changes over multiple generations. These are all crucial areas for future studies.

### Critical thermal maximum

The CTmax trial measures an acute response to thermal stress. The CTmax of juvenile Nile perch increased with acclimation temperature, suggesting that juvenile Nile perch have the capacity to adjust their thermal limits for short-term exposures to high temperatures. A review of temperature tolerances of North American freshwater fishes concluded that acclimation temperature shows a strong linear relationship to CTmin and CTmax (Beitinger *et al*., 2000). The slope relating juvenile Nile perch CTmax to acclimation temperature was of 0.30, which is within the range of values reported in the review (0.27–0.50). The authors add, however, that this linear relationship will hold true for acclimation temperatures within the range of temperature tolerance of the species, but not necessarily beyond this range. The highest temperature treatment of 29.5°C in the present study is a temperature occasionally encountered by Nile perch in their natural habitat, especially during the dry season (from February to April; Fig.1A). It is possible that Nile perch could reach a higher CTmax if acclimated to higher temperatures. Nevertheless, although the CTmax results suggest that the juvenile Nile perch has the capacity to adjust its thermal tolerance, the increase in CTmax (1.5°C) was less than the 4°C increase in acclimation temperatures, indicating that the thermal acclimation response does not keep pace with the absolute change in acclimation temperature.

### Effect of body size

Metabolic rates scale with body size, and there have been frequent attempts to generate a universal mass scaling exponent, *b*, for fishes, with diverse results. Vinberg (1960) reported 0.81 for freshwater fish. More recently, Clarke and Johnston (1999) suggested a scaling exponent of 0.79 for teleost fish based on a review of 138 studies on 69 species with values ranging from 0.40 to 1.29, whereas White *et al*. (2006) estimated its value at 0.88 based on results reported in the literature for 82 species. The scaling exponent for juvenile Nile perch derived from the regression of the log_10_-transformed relationship between RMR and body mass was 0.85. In an earlier study on the same species, Schofield and Chapman (2000) reported a scaling coefficient of 0.79 for individuals ranging between 8.0 and 12.0 cm TL.

Studies on the aerobic performance of fish at warmer temperatures are often conducted on fish of similar sizes. We did not proceed in the same way because we were interested in testing for differences in scaling of metabolic rates with body size across temperatures and natural habitats. If thermal sensitivity increases with body size because of hypothesized narrower thermal windows and lower optimal temperatures for growth (Pörtner and Knust, 2007; Pörtner *et al*., 2008), one could expect a steeper rise in RMR with body size for fish acclimated to warmer temperatures. Such a change could have explained the difference in mass exponent reported for Nile perch of 0.79 at 20°C (Schofield and Chapman, 2000) from the value of 0.85 in the present study. Our results do not provide evidence of increasing thermal sensitivity with body size in Nile perch, because the slope of the relationship between metabolic rate and body mass remained the same across temperatures. It is possible, however, that the body size range in the present study was not large enough to detect an increase in thermal sensitivity in larger Nile perch.

The CTmax of juvenile Nile perch was not correlated with body mass or TL, suggesting again that thermal sensitivity did not vary significantly with body size in this study. Similar results have been observed in Atlantic salmon (*Salmo salar*; Anttila *et al*., 2013), largemouth bass (*Micropterus salmoides*), Nile tilapia (*Oreochromis niloticus*), channel catfish (*Ictalurus punctatus*) and rainbow trout (*Oncorhynchus mykiss*; Recsetar *et al*., 2012). In contrast, Recsetar *et al*. (2012) observed that CTmax decreased slightly with TL for Apache trout (*Oncorhynchus gilae apache*) and Rio Grande cutthroat trout (*Oncorhynchus clarkii virginalis*).

### Effect of habitat

A number of studies have documented differences in metabolic rates between populations that reflect differences in their thermal regime. For example, evidence of local adaptation was found in two populations of *A. polyancanthus* experiencing different thermal regimes in their natural habitat (summer mean ± seasonal variation 27.0 ± 5.2 and 28.5 ± 5.3°C; Donelson and Munday, 2012). Both populations reached similar RMR when compared at their respective summer average temperature, +1.5 and +3.0°C treatments. Likewise, a northern population of *F. heteroclitus* showed a significantly higher RMR and MMR than a southern population, but similar AS when thermally acclimated to their experimental treatment, whereas acute trials generated mixed results (Healy and Schulte, 2012).

Although a large body of literature has documented intraspecific variation in a number of performance traits in response to warmer temperatures or hypoxia, very few studies have examined the effects of these combined stressors. Nonetheless, these two stressors can have interacting effects on thermal tolerance. In the context of the OCLTT framework, hypoxia is expected to limit MMR and thus AS and, because of the inverse relationship between oxygen solubility and temperature, thermal tolerance is expected to be reduced during exposure to hypoxia. However, adaptation to hypoxia may facilitate oxygen uptake in warmer waters and result in an increase in thermal tolerance (McBryan *et al*., 2013). For example, acclimation to hypoxia resulted in an increase in thermal tolerance in *I. punctatus* (Burleson and Silva, 2011). In their study of two subspecies of the common killifish (*F. heteroclitus*), McBryan *et al*. (2016) found that acclimation to warm waters increased both the time to loss of equilibrium in hypoxia and the total lamellar surface area of the gills as a result of regression of the interlamellar cell mass. The authors suggested that thermal acclimation may improve hypoxia tolerance by increasing gill surface area. An extensive study on the genetic basis of tolerance to temperature and hypoxia in *S. salar* revealed a positive correlation between thermal tolerance (CTmax) and hypoxia tolerance (Anttila *et al*., 2013). A potential explanation for this positive correlation is the fact that similar mechanisms are involved in tolerance to temperature and hypoxia (McBryan *et al*., 2016).

In the present study, we were interested in the potential effect of hypoxia tolerance on aerobic performance and upper thermal tolerance of Nile perch. Nile perch living in hypoxic conditions in their natural habitat should exhibit higher oxygen uptake capacity, which could enhance their thermal tolerance. The CTmax did not vary significantly between habitats, nor did AS, despite the contrasting oxygen regimes in both habitats. It is possible that acclimation time of Nile perch in normoxic water for the present study, although short, was sufficient for compensatory physiological changes to occur, thus affecting their hypoxia acclimation, which could explain the absence of significant differences between habitats.

Individuals from the wetland ecotone of Lake Nabugabo did not exhibit better aerobic performance, yet showed significantly larger gill size (Paterson *et al*., 2010). This divergence in gill size suggests phenotypic plasticity in response to hypoxic conditions or local adaptation. If juvenile Nile perch in wetland ecotones exhibit adaptive changes in response to hypoxia, one would predict a shift down in their metabolic machinery to lower basal metabolic maintenance costs, resulting in lower metabolic rates at all temperatures. Our results provide weak support for this prediction; we found a trend towards lower RMR for Nile perch from the wetland ecotone, whereas MMR did not differ between habitats. The divergence in gill traits in juvenile Nile perch is not maintained at the adult stage (Paterson *et al*., 2010), suggesting that it is an environmentally induced trait. Furthermore, Nile perch are likely to mix as adults in the pelagic zone, which reduces the possibility of a genetic component to these differences, although there is not yet empirical work to address this question.

### Conclusion

Understanding the ability of fish to adjust their thermal sensitivity and how close to their thermal limit they persist in their natural habitat are important factors to consider in the assessment of the impacts of predicted temperature increases associated with climate change (Stillman, 2003). Our CTmax trials demonstrated that juvenile Nile perch have the capacity to adjust their upper thermal limit with short-term acclimation to elevated temperatures. Juvenile Nile perch were also able to maintain large AS through the whole range of experimental temperatures, which could imply that the temperatures we tested were within their *T*_opt_ window. These findings suggest that Nile perch are not currently living at the edge of their thermal window in Lake Nabugabo, and provide evidence that these fish may adjust their thermal sensitivity through thermal acclimation, although not necessarily at a rate high enough to keep pace with the change in water temperature. Further studies are required to explore the fitness consequences of elevated temperatures on Nile perch.
